# Impact of intrauterine growth restriction on cerebral and renal oxygenation and perfusion during the first 3 days after birth

**DOI:** 10.1038/s41598-022-09199-5

**Published:** 2022-03-24

**Authors:** Paolo Montaldo, Simona Puzone, Elisabetta Caredda, Umberto Pugliese, Emanuela Inserra, Grazia Cirillo, Francesca Gicchino, Giuseppina Campana, Davide Ursi, Francesca Galdo, Margherita Internicola, Ferdinando Spagnuolo, Mauro Carpentieri, Carlo Capristo, Pierluigi Marzuillo, Emanuele Miraglia Del Giudice

**Affiliations:** 1 Department of Neonatology, University of Campania “Luigi Vanvitelli”, Naples, Italy; 2 Centre for Perinatal Neuroscience, Level 5 Hammersmith House, Imperial College London, Du Cane Road, London, W12 0HS UK

**Keywords:** Physiology, Cardiology

## Abstract

Intrauterine growth restriction (IUGR) is associated with a higher incidence of perinatal complications as well as cardiovascular and renal diseases later on. A better insight into the disease mechanisms underlying these sequalae is important in order to identify which IUGR infants are at a higher risk and find strategies to improve their outcome. In this prospective case–control study we examined whether IUGR had any effect on renal and cerebral perfusion and oxygen saturation in term neonates. We integrated near-infrared spectroscopy (NIRS), echocardiographic, Doppler and renal function data of 105 IUGR infants and 105 age/gender-matched controls. Cerebral and renal regional oxygen saturation values were measured by NIRS during the first 12 h after birth. Echocardiography alongside Doppler assessment of renal and anterior cerebral arteries were performed at 6, 24, 48 and 72 h of age. Glomerular and tubular functions were also assessed. We found a left ventricular dysfunction together with a higher cerebral oxygen saturation and perfusion values in the IUGR group. IUGR term infants showed a higher renal oxygen saturation and a reduced oxygen extraction together with a subclinical renal damage, as indicated by higher values of urinary neutrophil gelatinase-associated lipocalin and microalbumin. These data suggest that some of the haemodynamic changes present in growth-restricted foetuses may persist postnatally. The increased cerebral oxygenation may suggest an impaired transition to normal autoregulation as a consequence of intra-uterine chronic hypoxia. The higher renal oxygenation may reflect a reduced renal oxygen consumption due to a subclinical kidney damage.

## Introduction

Intrauterine growth restriction (IUGR) birth is defined as a foetal growth rate below its genetically determined potential size per race and gender and is associated with a higher risk of cardiovascular morbidity, mortality and long-term neurological adverse outcome^1,2^.

IUGR birth is mainly due to placental insufficiency, which can lead to chronic intrauterine hypoxia and prenatal haemodynamic disturbances, thus causing structural and functional changes of cerebral and renal circulation, which in turn may affect the renal and cardiovascular system in the long term^3–7^. In fact, chronic hypoxia, together with increased placental vascular resistance, may cause abnormal cardiac function due to the pressure and volume overload of the foetal heart, and determine a preferential redistribution of the blood flow to the brain, so as to maximize cerebral oxygenation^8^.

Previous studies demonstrated the presence of a cerebral vasodilation in small for gestational age (SGA) infants with changes in blood pressure and cerebral haemodynamic. This may reflect the extreme vulnerability of these neonates to perinatal hypoxia^9^. However, only few studies so far have assessed the relation between cerebral and systemic perfusion and oxygenation in the early postnatal period of growth-restricted term infants.

Near-infrared spectroscopy is a non-invasive tool for studying organ haemodynamic processes by measuring oxygenation and haemoglobin concentration changes. Functional echocardiography has been increasingly used to examine blood flow from the upper body, including the brain, thus providing a reliable assessment of systemic blood flow. The integration of these data may help to better understand the disease mechanisms responsible for the sequelae of IUGR and contribute to improve the identification of those IUGR neonates who are more likely to develop cardiovascular and renal diseases later on in life^1^.

We hypothesized that IUGR would affect postnatal cardiovascular transition and alter cardiac and renal functions in term infants, thus affecting brain and renal oxygenation. The primary outcome of the study was renal and cerebral tissue oxygen saturation through the application of near-infrared spectroscopy. Secondary outcomes were conventional echocardiographic parameters, cerebral and renal blood flow, glomerular and tubular function soon after birth.

## Methods

### Study design and population

This prospective case–control study was carried out in the post-natal ward of the Department of Neonatology, University of Campania “Luigi Vanvitelli”, Naples, Italy. We consecutively recruited SGA term neonates with intrauterine growth restriction (IUGR) and appropriate-for-gestational- age (AGA) controls born between January 2018 and January 2021. Each neonate with IUGR was matched to one neonate with AGA based on the age and gender. The Medical Ethical Committee of the University of Campania “Luigi Vanvitelli” approved the study and all procedures were performed in accordance with the relevant guidelines and regulations. Written informed consent was obtained from parents or legal representatives.

SGA was defined as birth weight < 10th centile for gestational age while the definition of adequate-for-gestational age was a birth weight between the 10th and the 90th percentile, both according to local percentile charts^10^. IUGR was defined according to the following criteria: estimated foetal birth weight below the 3rd percentile or below the 10th percentile^11^ in combination with one or more of the following parameters: abnormal Doppler waveforms in the umbilical (> 95th percentile), middle cerebral (< 5th percentile), or uterine (> 95th percentile) arteries and/or abnormal cerebroplacental ratio (< 5th centile)^12,13^. Only the last Doppler measurements prior to delivery were considered for the analysis.

Exclusion criteria were the need for additional oxygen or positive pressure ventilation at the time of birth, major congenital anomalies, genetic diseases, congenital heart disease, kidney, and metabolic diseases.

Antenatal, birth, maternal and neonatal characteristics were collected. As part of our hospital protocol all the IUGR neonates had capillary blood drawn three hours after birth to rule out hypoglycaemia. Therefore, parameters such as blood glucose, pCO_2_ and haemoglobin were only available for the IUGR group.

### Near infrared spectroscopy (NIRS) monitoring

Regional cerebral and renal oxygenations (rSO2) were studied continuously by multi-probe NIRS (ForeSight Elite, Casmed, Branford, CT) for the first 12 h after birth. A NIRS transducer was applied to the forehead for cerebral rSO2 measurement and secured with a bandage; another transducer was placed to the posterior flank at T12-L2 for renal rSO2. Proper transducer placement was verified concomitantly with renal ultrasound. Oxygen saturation of the blood (SpO2) and heart rate were measured simultaneously by pulse oximetry on the right hand (Nellcor pulse oximeter, Covidien, Boulder, CO, USA). Breast feeding and skin-to-skin care happened as usual without any interference from the research team.

Artefacts were considered as: (1) physiologically unexplained decrease or increase of at least 30% between two data points in rSO2 or other parameters, (2) changes in rSO2 combined with severely deformed accompanying variables, alternated with missing data points, together suggesting neonatal movement^14^. Fractional tissue oxygen extraction (FTOE) was calculated off-line with the formula: FTOE = (SpO2 − rSO2)/SpO2. After removing the artifacts, we considered tissue oxygenation data with a 1-h interval for the analysis.

### Echocardiographic and Doppler measurements

The echocardiographic and Doppler assessment was performed at 6, 24, 48 and 72 h of age using GE Logiq 7 (General Electric,USA) with 3–9 MHz transducer. Left Ventricular Cardiac Output (LVCO), Right Ventricular Cardiac Output (RVCO) and the superior vena cava (SVC) flow were calculated as previously described^15–17^. Resistance index in renal and anterior cerebral arteries were also assessed. A renal ultrasound of both kidneys was performed within 72 h after birth to measure renal length. Further details on the echocardiographic and Doppler measurements are in Supplementary file S1.

### Glomerular and tubular function

A blood sample was collected for serum creatinine and urea between 48 and 72 h after birth, at the same time as the neonatal metabolic screening. A paired urine sample was collected for microalbumin (Immunonephelometric method, BNTMII, Siemens Medical Solution) as well as a neutrophil gelatinase-associated lipocalin (NGAL) measurement (ELISA kit, Biorbyt LLC, St Louis, Missouri, MO, USA) by using single sterile bags according to manufacturer’s instructions. The detection limit for NGAL measurement was < 40 pg/mL.

Renal function was estimated according to the Schwartz formula for the term babies: estimated glomerular filtration rate (eGFR) = 0.45 × length/Serum Creatinine (measured by Jaffé’s method) in milligrams per deciliter.

### Statistical analysis

Continuous variables are presented either as means and SDs or median and interquartile range depending on their distribution. Categorical variables are given as numbers and proportions.

Differences in echocardiographic and Doppler measurements between IUGR and AGA newborns were assessed by using a Wilcoxon matched-pairs signed ranks test. We used a linear mixed-model analysis for NIRS parameters with individuals as a random effect nested within the groups and a fixed effect for the groups (IUGR versus AGA) and in order to assess whether the groups behaved differently over time. Results according to these linear mixed models are presented using means and 95% confidence intervals (CI). We also examined the effect of gender within the IUGR group by using a linear mixed model with a fixed effect for time and sex (male vs. female). A first-order autoregressive moving average covariance structure was used in these analyses. Statistical analyses were performed using SPSS Statistics V.24 (IBM). A p value < 0.05 was considered as significant.

We estimated a sample size of 85 patients for each group, based on the following assumptions: (i) the difference in the cerebral or renal rSO2 between the IUGR and control group would have to be at least 3%, (ii) SD for cerebral and renal rSO2 in control newborns is 8% and 6% respectively^18^, (iii) a two-sided test of statistical significance, (iv) a probability of 0.05 for a type I error associated with the two-sided test, and (v) a probability of 0.1 for a type II error associated with the test (i.e., the power of the test is 90%), (vi) the sample size has been increased of 100 patients in each group to take into account missing data.

## Results

### Study flow and baseline variables

**Figure 1:**
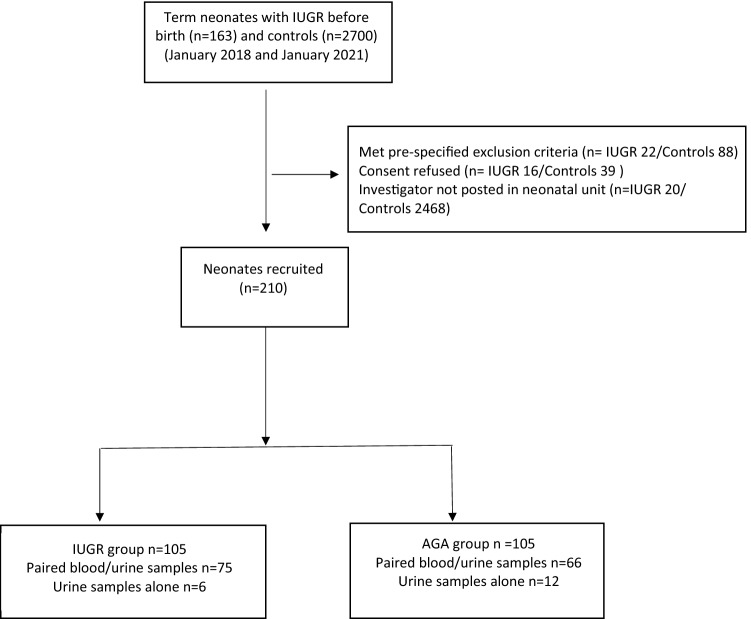
Flowchart of patient selection.

Two-hundred-ten neonates were recruited in the study period, 105 of whom were SGA and 105 AGA term neonates (Fig. 1).

Birth weight, length and cranial circumference were significantly lower in the IUGR group (Table 1). SpO2 and heart rate were not significantly different between the two groups (SpO2 main effect group: p = 0.57; interaction time × group: p = 0.43) (heart rate main effect group: p = 0.764; interaction time × group: p = 0.89) over the whole observation period. Within the IUGR group, there was no difference between male and female neonates in SpO2 (interaction time × sex: p = 0.32; main effect sex: p = 0.21).

**Table 1:** Baseline maternal and neonatal characteristics in the IUGR and control groups.

Variables	Control group (n = 105)	IUGR group (n = 105)	P value
Gestational age, weeks, mean (SD)	39 (1.5)	39(1.6)	0.49
Primigravida, n (%)	47 (45)	46 (44)	0.89
Diabetes, n (%)	4 (4)	8 (7.6)	0.23
Hypertension, n (%)	12(11.4)	28 (26.7)	0.008*
Antibiotics during Pregnancy, n (%)	11 (10)	14 (13)	0.51
C section, n (%)	35 (33.3)	30 (28.6)	0.55
Female, n (%)	55(52)	55(52)	-
Birth weight, g, median [IQR]	3180 [3000–3400]	2640 [2400–2780]	0.001*
Birth length, cm, median [IQR]	51 [50–53]	48 [47–49]	0.001*
Birth head circumference, cm, median [IQR]	34.7 [33.8–35]	33 [32–34]	0.001*
Apgar score 1 min, median [IQR]	9 [8.5–9]	9 [8–9]	0.77
Apgar score 5 min, median [IQR]	10 [9–10]	9 [9–10]	0.21
Cord blood pH, median [IQR]	7.29 [7.23–7.31]	7.31 [7.25–7.4]	0.11
Haemoglobin, g/dL, mean (SD)	NA	19.1 (2.4)	–
pCO2, mmHg, mean (SD)	NA	50.8 (5.9)	–
Blood glucose, mg/dL, mean (SD)	NA	72.6 (29.7)	–
Serum Cr, mg/dL, mean (SD)	0.57 (0.11)	0.69 (0.10)	0.13
Serum urea, mg/dL, mean (SD)	16.2 (7.7)	17.1 (2.0)	0.71

Mann–Whitney U test was used for nonparametric data, Student’s t test for parametric data, and the χ^2^ test for categorical measurements.

*Statistically significant (P < 0.05).

### Primary outcome

#### Renal and cerebral rSO2 and FTOE

**Figure 2:**
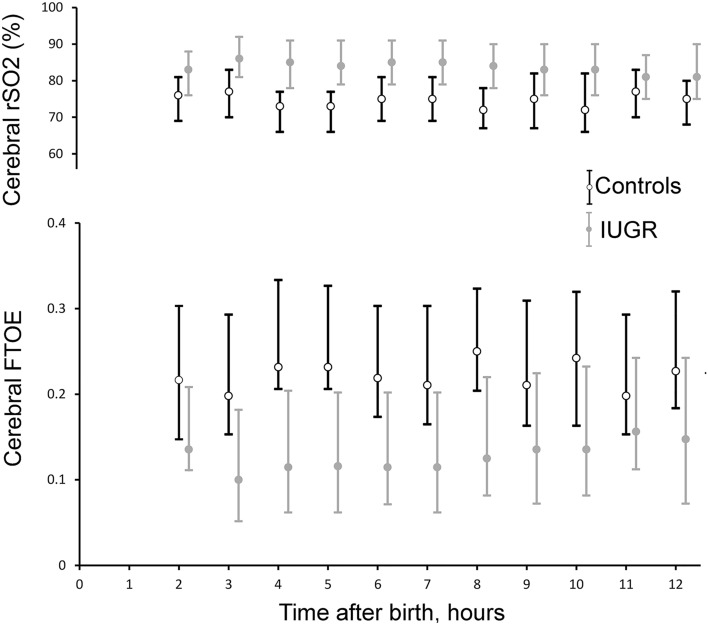
Cerebral regional oxygen saturation (rSO2) and fractional tissue oxygen extraction (FTOE) values recorded during the first 12 h after birth in intrauterine growth restriction (IUGR) (n = 105) and control (n = 105) newborns. Grey filled circles represent IUGR and black hollow circles control infants. Data are given as mean (circles) and standard deviation (error bar). There was a significant group difference, but the groups did not behave differently over time.

NIRS monitoring started at a mean time of 68 min ± 22 min. Therefore, NIRS values are reported from the second hour after birth on. There were significantly higher cerebral rSO2 (main effect group: p = 0.04; interaction time × group: p = 0.72) and lower FTOE values in the IUGR compared with the control group (main effect group: p = 0.03; interaction time × group: p = 0.463) over the whole observation period (Fig. 2).

**Figure 3:**
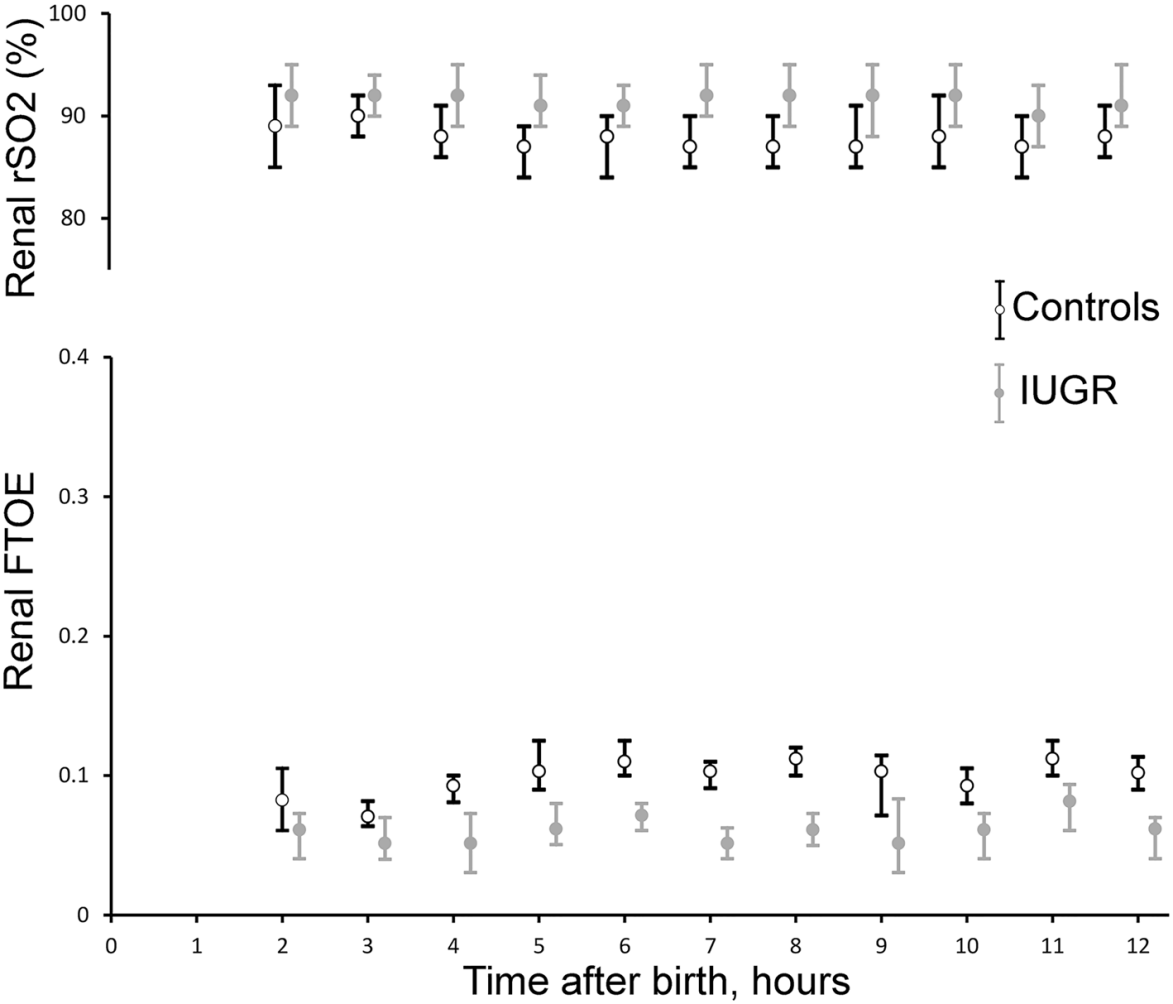
Renal regional oxygen saturation (rSO2) and fractional tissue oxygen extraction (FTOE) values recorded during the first 12 h after birth in intrauterine growth restriction (IUGR) (n = 105) and control (n = 105) newborns. Grey filled circles represent IUGR and black hollow circles control infants. Data are given as mean (circles) and standard deviation (error bar). There was a significant group difference, but the groups did not behave differently over time.

Renal FTOE was significantly lower in IUGR versus control neonates (main effect group: p = 0.04; interaction time × group: p = 0.39) whereas renal rSO2 was higher in IUGR versus control neonates (main effect group: p = 0.003; interaction time × group: p = 0.44) (Fig. 3). There was no significant correlation between haemoglobin concentration and renal and cerebral rSO2 at the different time points in the IUGR group (p > 0.05).

### Secondary outcomes

#### Echocardiography and Doppler measurements

**Figure 4:**
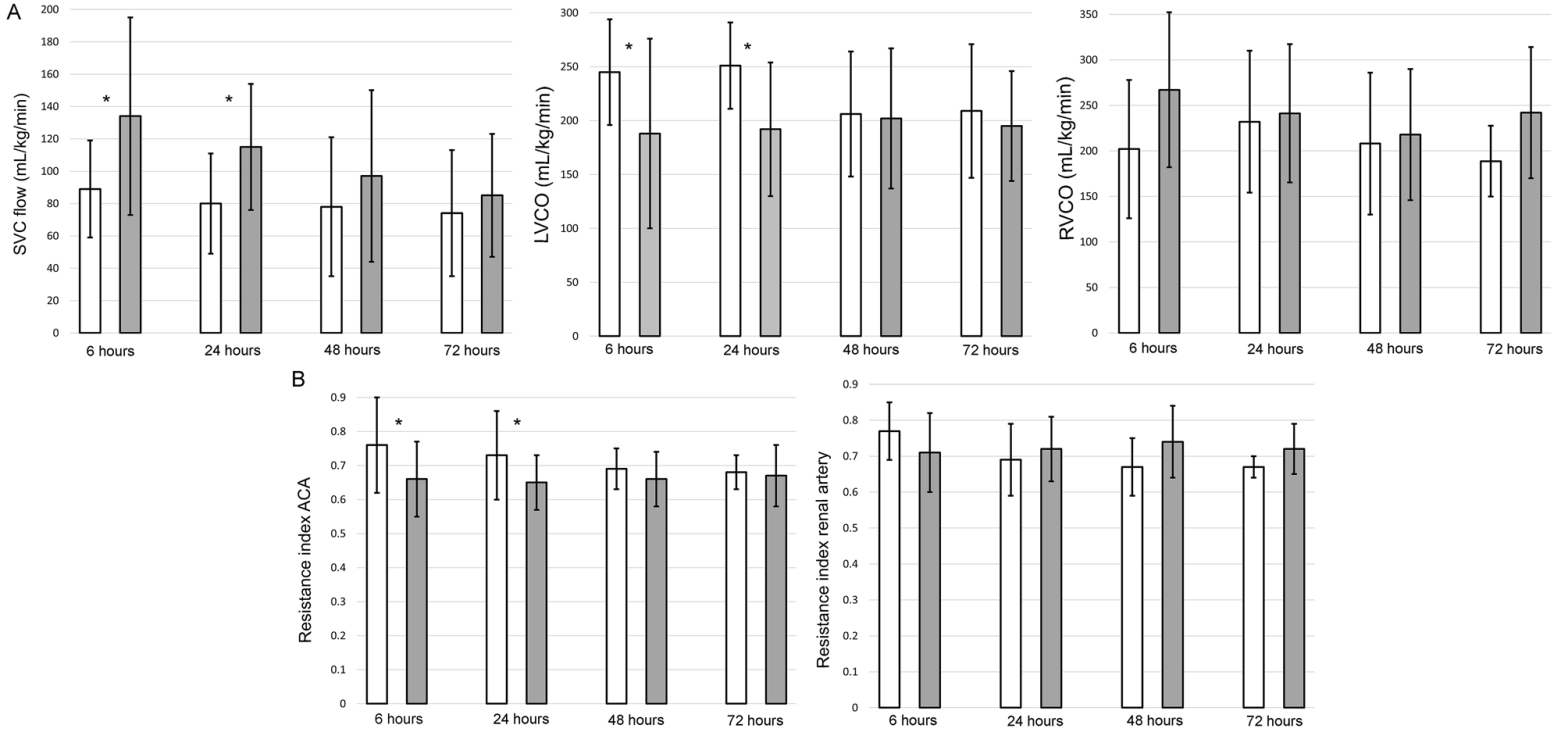
Bar graph showing the mean echocardiography (**A**) and Doppler measurements (**B**) in intrauterine growth restriction (IUGR) (grey) and control (white) newborns at the different time points. Echocardiography and Doppler assessments were not performed in eight infants in the control and three infants in the IUGR group at 72 h. Error bars represent standard deviation. *Statistically significant (P < 0.05). *SVC* superior vena cava, *LVCO* left ventricular cardiac output, *RVCO* right ventricular cardiac output, *ACA* anterior cerebral artery.

The SVC flow at 6 and 24 h after birth were significantly higher in IUGR than in the control infants (134 ± 61 versus 89 ± 30 ml/kg/min, 6 h p = 0.02; 115 ± 39 versus 80 ± 31 ml/kg/min, 24 h p = 0.0001). After 24 h SVC flow was not significantly different between IUGR and control infants (97 ± 53 versus 78 ± 43 ml/kg/min, 48 h p = 0.48; 85 ± 38 versus 74 ± 39 ml/kg/min, 72 h p = 0.50). LVCO was significantly lower in IUGR than in control infants at 6 and 24 h after birth (188 ± 88 versus 245 ± 49 ml/kg/min, 6 h p = 0.03; 192 ± 62 versus 251 ± 40 ml/kg/min at 24 h, p = 0.004). After 24 h LVCO was not significantly different between IUGR and control infants (202 ± 65 versus 206 ± 58 ml/kg/min, 48 h p = 0.92; 195 ± 51 versus 209 ± 62 ml/kg/min, 72 h p = 0.71). RVCO was not statistically different between IUGR and control infants at all the time points (267.1 ± 85 versus 201 ± 76 ml/kg/min, 6 h p = 0.06; 241.2 ± 76 versus 232 ± 78 ml/kg/min, 24 h p = 0.64; 217.8 ± 72 versus 208 ± 78 ml/kg/min, 48 h p = 0.75; 242 ± 72 versus 188.7 ± 39 ml/kg/min, 72 h p = 0.09) (Fig. 4A).

A small patent ductus arteriosus (PDA) closed spontaneously in the majority of neonates by the second day. A PDA was present in 97 out of 105 (92%) in the IUGR versus 94/105 (89%) infants in the control group at 6 h (p = 0.63). At 24 h 46/105 (44%) in the IUGR versus 49/105(47%) infants in the control group had a PDA (p = 0.78). Six out of 105 (6%) in the IUGR versus 4/105 (4%) infants in the control group still had a PDA at 48 h (p = 0.74). At 72 h 2/105 (2%) in the IUGR versus 3/105 (3%) infants in the control group had a PDA (p = 0.65).

The resistance index of the anterior cerebral artery was significantly lower in the IUGR group at 6 and 24 h (0.66 ± 0.11 versus 0.76 ± 0.14, 6 h p = 0.007; 0.65 ± 0.08 versus 0.73 ± 0.13, 24 h p = 0.04). At 48 and 72 h after birth, resistance index of the anterior cerebral artery was not significantly different between IUGR and control infants (0.66 ± 0.08 versus 0.69 ± 0.06, 48 h p = 0.46; 0.67 ± 0.09 versus 0.68 ± 0.05, 72 h p = 0.87). The resistance index of the renal artery was not statistically different between IUGR and control infants at the different time points (0.71 ± 0.11 versus 0.77 ± 0.08, 6 h p = 0.16; 0.72 ± 0.09 versus 0.69 ± 0.10, 24 h p = 0.76; 0.74 ± 0.10 versus 0.67 ± 0.08, 48 h p = 0.18; 0.72 ± 0.07 versus 0.67 ± 0.03, 72 h p = 0.14) (Fig. 4B).

#### Glomerular and tubular function

**Figure 5:**
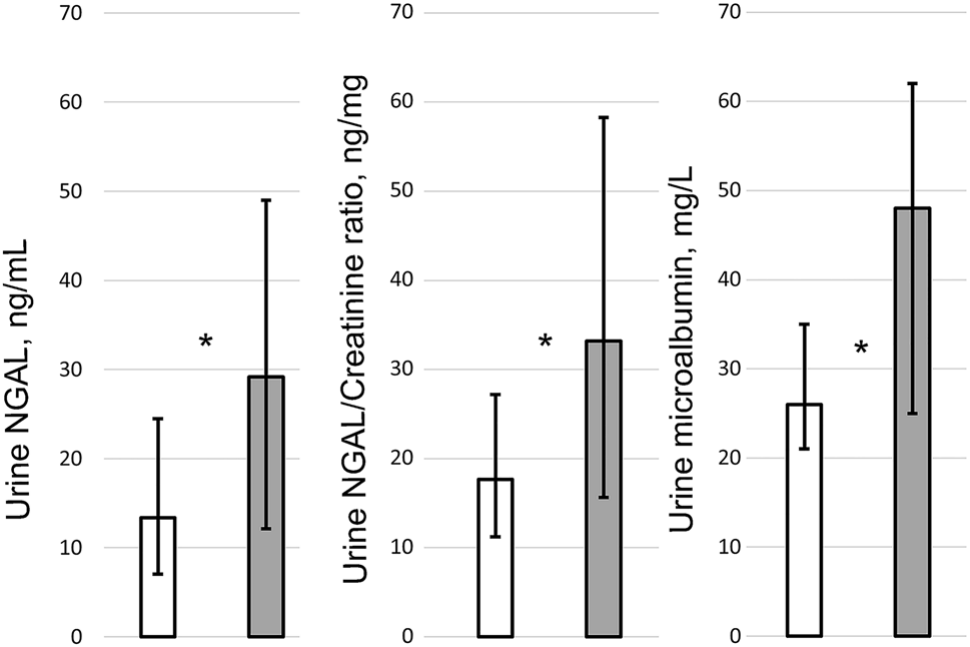
Bar graph showing the median urinary neutrophil gelatinase-associated lipocalin (NGAL), NGAL/Creatinine and urine microalbumin values in intrauterine growth restriction (IUGR) (grey) and control (white) newborns. Error bars represent the upper and lower quartiles. Paired blood/urine samples were available in 75 IUGR and 66 control newborns. Urine samples alone were available in 6 IUGR and 12 control infants. *Statistically significant (P < 0.05).

One-hundred-fifty-nine urine and 141 blood samples were available for analysis (Fig. 1). Urine microalbumin and NGAL levels were significantly higher in IUGR compared with control infants (Urine NGAL 29.16 [IQR12.10–49.01] versus 13.36 [IQR 7.04–24.45] ng/mL, p = 0.04; urine NGAL/Creatinine ratio 33.2 [IQR 15.61–58.26] versus 17.69 [IQR 11.24–27.16] ng/mg creatinine, p = 0.04; urine microalbumin 48 [IQR 25–62] versus 26 [IQR 21–35] mg/L, p = 0.01) (Fig. 5).

The eGFR was not significantly different between IUGR and AGA infants (36.5 ± 11.52 versus 40.6 ± 9.14 mL/min/1.73 m^2^, p = 0.28). There was no statistically significant difference in the kidney size between IUGR and AGA neonates (right kidney 40 [IQR 38–43] versus 42 [IQR 40–44] mm, p = 0.13, left kidney 40.5 [IQR 37–43] versus 45 [IQR 42–47] mm, p = 0.24).

## Discussion

Our study shows that IUGR term infants have an increased cerebral oxygenation and perfusion during the first day after birth, as indicated by a higher cerebral rSO2 and SVC flow values. This may reflect a persistence of the abnormal cerebral blood flow features present in growth-restricted foetuses. We also found a higher tissue oxygenation in the kidney without any significant difference in the renal blood flow.

Chronic hypoxia causes extensive haemodynamic changes in growth-restricted foetuses so as to preserve brain oxygen supply and protect the foetal brain in an unfavourable intrauterine environment. These changes include a decrease in the cerebral vascular resistance due to a local increase of adenosine, nitric oxide and prostanoids^19^. In agreement with us, other researchers found that these intrauterine haemodynamic changes may persist during the early neonatal period. In fact, significant changes in the middle and anterior cerebral artery resistive and pulsatility indices have been found in IUGR neonates in the first few days of age^9,20,21^. Whether this altered brain blood flow and oxygenation themselves contribute to a higher vulnerability in the brain growth, has been the focus of different studies. Previous research has hypothesized that the postnatal continuation of the increased cerebral blood flow might cause hyperoxia, which may trigger a burst of free reactive oxygen species^22–24^. This long-lasting oxidative stress and cell damage to oxygen-sensitive organs such as the brain may contribute to the neurological damage^24^. Of note, postnatal changes in cerebral blood flow velocity have been suggested as prognostic markers for adverse neurodevelopment in IUGR neonates^25^.

Our finding of higher cerebral rSO2 values in IUGR term neonates is in accordance with cerebral oxygenation data in IUGR preterm infants where higher values have been consistently reported in the first days after birth^26,27^. Recently, Baik-Schneditz et al. also showed significantly higher cerebral rSO2 values and lower rates of oxygen extraction during the first 15 min after birth in IUGR preterm and term neonates when compared with AGA peers^28^. In regard to cerebral oxygen extraction, we found that IUGR infants had a decreased cerebral FTOE after birth. This decreased cerebral oxygen consumption may be explained as the persistence of an adaptation phenomenon due to the reduced substrate delivery in case of foetal chronic hypoxia. This is supported by animal models, which indicate that chronic foetal hypoxia ultimately reduces oxygen consumption in order to decrease substrate requirements for energy and growth^29,30^. This ability during foetal life has been shown to be critical to reduce the loss of white and grey matter volume in IUGR infants^31^.

To date there is a paucity of data regarding the postnatal transition of IUGR term neonates and in many instances, there are no echocardiography data or simultaneous evaluation of peripheral circulation or oxygenation.

Our study pointed out a compromised systolic function, which may reflect the increased cardiac afterload due to the high placental resistance during the foetal life. Growth-restricted foetuses have been found to have an impaired systolic function as indicated by decreased peak systolic velocities in the cardiac outflow tracts and a diminished ejection force^32,33^. However, there are conflicting results so far regarding whether a systolic dysfunction occurs also in the early postnatal period in IUGR infants, which highlights the heterogeneity of this population. In fact, while most of the studies performed in preterm IUGR infants reported a similar cardiac output in IUGR neonates and controls^34–38^, a systolic dysfunction soon after birth has been found in IUGR term infants^39^. These findings are likely to reflect the cardiac adaptation to prolonged IUGR. Unfortunately, we did not assess the intestinal blood flow. Therefore, we could not examine whether the decreased LVCO and higher renal rSO2 were related to a decreased intestinal with a preserved renal blood flow.

A better insight into how growth-restricted neonates utilize oxygen may help to understand the later development of hypertension and chronic kidney disease. Only few studies have examined the renal rSO2 of IUGR neonates so far^40^. Terstappen et al. assessed renal and cerebral rSO2 in 9 growth-restricted and 7 control preterm neonates and showed a higher cerebral and renal tissue oxygenation. Similar to our study, renal artery blood flow was not different when compared to controls suggesting that oxygenation differences were due to underlying renal development or physiology^41^. This is supported by animal studies and antenatal Doppler data, which suggested that mild hypoxemia does not affect foetal renal blood flow or urine production rate directly, but indirectly due to a decreased renal growth^42–44^. Another research showed that neonates with an early diagnosis of growth restriction during pregnancy had a higher renal FTOE after birth compared to those with a later diagnosis^45^.

We hypothesize that the higher renal tissue oxygenation without any significant difference in the renal blood flow may indicate lower oxygen consumption. Renal oxygen delivery is determined by three major factors which are renal blood flow, local tissue perfusion and blood oxygen content whereas oxygen consumption is mainly affected by glomerular filtration rate and sodium reabsorption^46^. Our findings of higher NGAL and urine microalbumin levels in IUGR neonates indicate a subclinical kidney damage after growth restriction. NGAL is a protein produced by epithelial and neutrophils cells and represents a direct marker of tubular damage. Proximal tubules cells are extremely vulnerable to hypoxia. Therefore, in case of chronic intrauterine hypoxia such as placental insufficiency, these cells are likely to be affected. Previous research also reported that growth-restricted infants have subclinical kidney damage, as showed by significantly higher urinary NGAL and urinary NGAL /Creatinine^47^.

Renal dysfunction is often due to impaired vascular tone and integrity after IUGR^48^. Increased renal oxygenation itself is closely linked to oxidative stress, which can trigger renal afferent arterioles vasoconstriction and myogenic response enhancement thus further promoting hypertension and nephropathy later in life^49^.

Our study has strengths and limitations. Firstly, this study integrates serial data from echocardiography, Doppler and NIRS, thus providing further insights into the haemodynamic changes in IUGR infants during the first days after birth. IUGR neonates are a heterogeneous population with timing, severity and duration of hypoxaemia playing a role in the cardiovascular transition^50^. Term IUGR infants are often delivered after milder forms of placental insufficiency and most cases have a late onset growth restriction. In contrast, the early onset growth-restricted fetus is more likely to be delivered preterm^50^. We used stringent inclusion criteria to avoid any potential confounder due to this heterogeneity. The recruitment of other categories of neonates like preterm or term neonates requiring delivery room resuscitation, would have made it difficult to understand the contribution of each of these factors to the cerebral and renal oxygenation/perfusion without a much larger sample size. Secondly, we correlated NIRS parameters at birth with Doppler and renal function measurements so as to estimate renal oxygen consumption in IURG infants. Furthermore, we used also early markers of kidney damage like urinary NGAL and microalbumin. The main limitation of our study is that we did not assess the intestinal blood flow. Therefore, we could not explore the relation among decreased LVCO, renal and intestinal blood flow. In addition, there is a lack of data from the immediate transition period and a limited duration of NIRS monitoring (12 h). Finally, data on the renal function were available in only a subset of infants.

## Conclusions

This study shows that IUGR has a direct impact on the cerebral and renal oxygenation during the first days after birth in term infants. The increased renal oxygenation in IUGR neonates may be due to an impaired subclinical renal function with reduced oxygen consumption. The increased cerebral oxygenation may suggest an impaired transition to normal auto-regulation as a consequence of intra-uterine chronic hypoxia. Whether these observations correlate with short and long-term outcomes, it needs to be investigated in longitudinal studies with an adequate follow-up.

## Supplementary Information

Supplementary Information.

## Data Availability

Data are available upon reasonable request and once all the different sub-studies have been published.
