# Supplementary material for: Impact of intrauterine growth restriction on cerebral and renal oxygenation and perfusion during the first 3 days after birth
Source: Sci Rep. 2022 Mar 24;12:5067. doi: 10.1038/s41598-022-09199-5 (PMC8948256; doi:10.1038/s41598-022-09199-5)
Supplement: Supplementary file 1 — Supplementary Information. [file 41598_2022_9199_MOESM1_ESM.pdf]

Supplementary file 1

Further details on the echocardiographic, Doppler assessment.

Echocardiography and Doppler assessment

Neonates were placed in cradles and were studied when quiet or asleep. Left Ventricular Cardiac Output (LVCO) was calculated by multiplying the stroke volume using the aortic valve diameter in a mid-parasternal long-axis 2D view, the velocity time interval measured by pulsed- wave Doppler in an apical 5- chamber view and the heart rate (10). Right Ventricular Cardiac Output (RVCO) was calculated by using the same formula as LVCO (10). Right ventricular outflow tract diameter was measured from the tilted parasternal long-axis view at the pulmonary valve insertion level and the velocity time interval was acquired in the same view (9-10). LVCO and RVCO were averaged over 3 consecutive heart cycles (10). Superior vena cava (SVC) flow velocity was measured from the low subcostal view and the pulsed Doppler recording was made at the junction of the SVC and the right atrium. The SVC diameter was measured through the high parasternal long axis view with the beam in a true sagittal plane. The maximum and minimum internal diameters were measured and averaged from three to five cardiac cycles. Mean velocity of blood flow was calculated from the integral of the Doppler velocity tracings and was averaged over 10 consecutive heart cycles (11).

Anterior cerebral artery and renal artery blood flow were visualized with colour and pulsed Doppler in sagittal view through the anterior fontanel and lateral view in the supine or lateral decubitus position. The peak systolic velocity (PSV) and end-diastolic velocity (EDV) of Doppler waveforms were measured and subsequently used to calculate resistance index using the equation:

Resistance index = PSV − EDV/PSV.

The mean resistance index was calculated on 3 sequential waveforms.
